# Black:white inequities in infant mortality across the 69 most populous US cities, 2018–2021

**DOI:** 10.3389/fpubh.2025.1484433

**Published:** 2025-02-26

**Authors:** Nazia S. Saiyed, Jessica C. Bishop-Royse, Britney P. Smart, Anne Leung, Maureen R. Benjamins

**Affiliations:** ^1^Sinai Urban Health Institute, Sinai Chicago, Chicago, IL, United States; ^2^College of Nursing, Rush University, Chicago, IL, United States; ^3^Chicago Medical School, Rosalind Franklin University of Medicine and Science, North Chicago, IL, United States

**Keywords:** infant mortality, birth outcomes, racism, structural racism, inequities, disparities, health equity, urban

## Abstract

The United States has poor birth outcomes, including high rates of infant mortality and substantial racial inequities, compared to other developed nations. However, both overall mortality rates and racial inequities in rates vary across locations, emphasizing the structural forces that shape population health. We used mortality and natality data from the National Vital Statistics System to assess racial inequities in infant mortality rates within the most populous US cities for 2018–2021. Specifically, we: (1) calculate overall and race-specific infant mortality rates for 69 cities and racial inequities in infant mortality for 48 cities; and, (2) analyze associations between these inequities and city-level measures of structural racism. City-level infant mortality rates ranged from 1.96 deaths per 1,000 births in Irvine, CA to 13.92 in Detroit, MI. The non-Hispanic Black infant mortality rate was 2.5 times higher than the non-Hispanic white rate in the US and the Black:white rate ratio was statistically significant in all study cities, ranging from 1.8 (Omaha, NE) to 5.0 (Cincinnati, OH). The Black:white rate ratio was greater than 4.0 in 10 cities. Overall and race-specific infant mortality rates were associated with measures of education, economic status, incarceration, segregation, and diversity. Racial inequities in infant mortality were associated with measures of economic status. Understanding infant mortality inequities at the city level is critical to support the efforts of urban health advocates. Moreover, examining the persistent associations of structural racism with infant mortality will help guide necessary programmatic or policy decisions to reduce infant mortality in US cities.

## Introduction

Infant mortality rates in the United States (US) compare poorly to those rates seen in peer countries and vary substantially among racial and ethnic groups. While US infant mortality rates declined substantially over the past two centuries, they increased slightly in 2022, to 5.6 deaths per 1,000 births, the highest among the most economically developed countries. Moreover, structural and social factors have led to the Black infant mortality rate (10.9/1,000) being two to three times larger than that of other racial and ethnic groups (1). This racial gap in infant mortality is not consistent across the country; there are larger disparities in midwestern and southern states compared to western regions (2). Data from 2013 to 2015 revealed wide variation in state-level race-specific infant mortality rates, with white infant mortality rates ranging from 2.5 (District of Columbia) to 7.0 (Arkansas), while Black infant mortality rates during the same period ranged from 8.3 (Massachusetts) to 14.3 (Wisconsin) (3).

Structural racism has been defined as the “totality of ways in which societies foster racial discrimination through mutually reinforcing systems of housing, education, employment, earnings, benefits, credit, media, health care, and criminal justice” [(4), p. 1453]. Racial inequities in infant mortality and other health outcomes are driven by structural racism, which limits the opportunities, resources, power, and well-being of communities of color (4). Some common domains used to define and measure structural racism include: education, employment, transportation, residential neighborhoods and housing, immigration and border enforcement, political participation, socioeconomic status, criminal justice, and workplace environment (5–8).

The impact of structural racism on infant mortality has been demonstrated at a variety of geographic levels in the US, including states, counties, and neighborhoods or community areas. At the state level, Wallace et al. found larger median household income, more highly educated residents, and higher rates of engagement with professional employment were associated with lower infant mortality (2017). A number of measures have been linked specifically to Black infant mortality rates, including inequities in education, employment, and the judicial system (9–11). Vilda et al. found that racial inequities in infant mortality increased in urban counties with a higher composite structural racism score (2021). At a smaller geographic level, Bishop-Royse et al. found an association between the Index of Concentration at the Extremes, a measure that represents the concentration of disadvantage in a geographic area, and infant mortality rates in Chicago community areas, which persisted after adjusting for socio-economic marginalization, hardship, household composition and family support, and healthcare access (2021).

Given the increased understanding of the role of structural racism in inequities in infant mortality and other adverse birth outcomes, the Centers for Disease for Control and Prevention began supporting a shift in focus from interventions addressing individual-level indicators to those that address more systemic and social factors in the 1990s, along with improved research and greater community involvement. Many interventions have been developed to address poor maternal and child health in cities and states across the US; while some such interventions have shown meaningful improvements or promise, inequities in infant mortality have remained high (12, 13).

Population-focused interventions need to be guided by population-level data. A number of research groups have explored city-level data on infant mortality and other poor birth outcomes, including low birthweight, preterm birth, teen births, and prenatal care [Big Cities Health (14, 15)]. However, data that explicitly compares infant mortality rates for non-Hispanic Black and non-Hispanic white populations and data that examines infant mortality rates in relation to city-level measures of structural racism and socio-demographic characteristics is missing. Given the geographic variation in inequities in infant mortality and the persistence of those inequities over time, an understanding of infant mortality rates and inequities at the city level is crucial to better understand factors that might contribute to poorer birth outcomes and support the efforts of urban health advocates. Moreover, assessing the association of structural racism with infant deaths will help guide necessary programmatic or policy decisions to improve infant mortality in US cities. The current study compares infant mortality rates from 2018 to 2021 for the non-Hispanic Black and non-Hispanic white populations and explores associated city-level risk factors within the 69 most populous US cities.

## Methods

This study was ruled exempt from review by the Mount Sinai Hospital institutional review board because it uses deidentified data. The study followed the Strengthening the Reporting of Observational Studies in Epidemiology (STROBE) reporting guidelines.

### Study population

The analysis was limited to cities with total populations of at least 300,000 people in the 2020 Census. Cities with smaller populations were excluded due to their likelihood of having fewer than 20 infant deaths during the study period. County data were used in place of city data in five locations where city and county governments have consolidated and city-specific natality and mortality data are not available (Nashville and Davidson County, TN; Indianapolis and Marion County, IN; Louisville and Jefferson County, KY; Honolulu and Honolulu County, HI; and Lexington and Fayette County, KY). The 69 included cites make up 17% of the US population. Demographic and socioeconomic characteristics of people residing in the cities are described in Supplementary Table 1.

### Data sources

Mortality data was drawn from the “Detailed Mortality—All Counties” restricted use data files for 2018–2021 from the National Vital Statistics System (NVSS). We extracted records for all deaths of infants who were less than 1 year of age by race, ethnicity, and place of residence. We excluded the records of non-US residents and records in which infant age was missing. Death certificate data, including race and ethnicity, are completed by proxy (e.g., funeral director or attending physician). Natality data was drawn from the “Natality—All Counties” restricted use data files for 2018–2021 from NVSS. Records for all births were extracted by the race, ethnicity, and place of residence of the mother.

We examined associations between city-level infant mortality rates and a composite measure of structural racism, several indexes that capture racial and ethnic inequity, and a number of unidimensional socio-economic characteristics summarized at the city level. From the 2020 US Census and 2017–2021 American Community Survey 5-year estimates, we included high school graduation rate (overall, Black, and white), poverty rate (overall, Black, and white), median household income (overall, Black, and white), percent uninsured (overall, Black, and white), percent of households with very high rent burden (>35% of income), percent of adults age 18–64 housed in correctional facilities (overall, Black, and white), and Gini coefficient, a measure of income inequality, for each city. Measures of neighborhood racial and ethnic segregation and racial and ethnic diversity for all of the cities came from the City Health Dashboard (15). Finally, we incorporated a measure of structural racism, developed by Siegel et al. using latent class analysis and structural equation modeling (27). This measure combines 14 different variables on 5 dimensions of structural racism (segregation, employment, economic status, education, and incarceration) into a single measure expressed in terms of standard deviations from the mean for all cities (which was represented by zero).

### Statistical analysis

Overall, non-Hispanic Black, and non-Hispanic white infant mortality rates were calculated for the US as a whole and for each of the cities included in the study. Race-specific mortality rates (hereafter referred to as Black and white) and measures of inequity were calculated for all cities and city sub-groups where there were 20 or more infant deaths. A four-year average infant mortality rate (2018–2021) was calculated to mitigate volatility in estimates due to low numbers of infant deaths in any single year. Infant mortality rates were calculated by dividing the total number of infant deaths during the period from 2018 to 2021 by the number of live births in the same period. Infant mortality rates are reported per 1,000 births.

Inequities between Black and white populations were described using rate ratios, rate differences, and annual excess Black deaths. Excess Black deaths were calculated by determining how many Black infants would have died if the Black population experienced the same infant mortality rate as the white population in each city, then subtracting the expected number of deaths from the observed deaths for each year.

We used Spearman’s Rank Correlation to assess the presence of significant correlations between four key outcomes (overall infant mortality rate, Black infant mortality rate, white infant mortality rate, and Black:white infant mortality rate ratios) and each of the aforementioned measures of city-level sociodemographic characteristics, segregation, diversity, and structural racism. Significance was set at *p* < 0.05.

Analysis for this study was completed using SAS version 9.4 and Stata version 18.1.

## Results

Our analysis included 81,890 infant deaths and 14,817,191 births during the 4-year study period. The US and each of the 69 cities had at least 20 infant deaths among the total population for the study period. Non-Hispanic Black infant mortality was not calculated for 15 cities where there were fewer than 20 deaths to Black infants during the study period. Similarly, non-Hispanic white infant mortality rates were not calculated in 13 cities for the same reason. In 48 cities and the US where there were at least 20 infant deaths in both the Black and white populations, we calculated Black:white infant mortality rate ratios, Black:white infant mortality rate differences, and excess Black infant deaths.

### Overall and race-specific infant mortality

**Figure 1:**
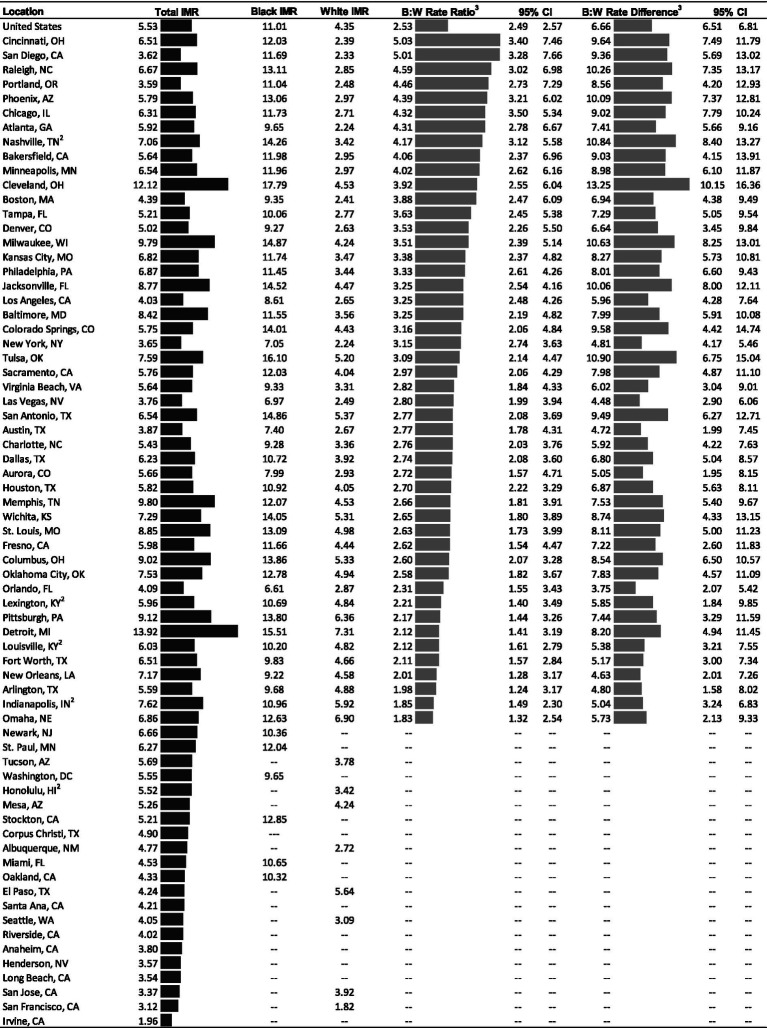
Age-adjusted infant mortality rates^1^ and measures of inequities (2018–2021). Notes: IMR = infant mortality rate; NH = non-Hispanic; B:W = Black:white; CI = confidence interval. ^1^Mortality rates are shown per 1,000 births. ^2^Data is for consolidated city-county. ^3^All rate ratios and rate differences are significant (*p* < .05). “---” Indicates data were suppressed due to a low number of infant deaths during the study period.

The infant mortality rate for the US in 2018–2021 was 5.53 deaths per 1,000 births (see Figure 1). The city-level rates ranged from 1.96 in Irvine to 13.92 in Detroit. Of the 69 cities, 41 cities had higher infant mortality rates than the US and 28 had lower rates. The Black infant mortality rate for the US was 11.01. Black infant mortality rates in study cities ranged from 6.61 in Orlando to 17.79 in Cleveland. Thirty of the 54 cities where Black rates could be calculated had rates higher than the US rate (11.01); three cities had rates over 15 deaths per 1,000 births (Detroit, Tulsa, and Cleveland). The white infant mortality rate for the US was 4.35. The city-level white rate ranged from 1.82 in San Francisco to 7.31 in Detroit. Of the cities where white infant mortality rates were calculated, 21 of 56 had rates greater than the US rate.

### Inequities in infant mortality

Strikingly, the Black infant mortality rate was higher than the white infant mortality rate in the US and all 48 cities with sufficient data for both groups. The rate ratio, which represents the relative difference in Black and white infant mortality was 2.53 for the US. In other words, the Black infant mortality rate was 2.53 times higher than the white rate during the study period. Among cities, the Black:white rate ratio ranged from 1.83 in Omaha to 5.03 in Cincinnati. Moreover, the rate ratio was greater than 4 in 10 cities and greater than five in two (San Diego and Cincinnati).

**Figure 2:**
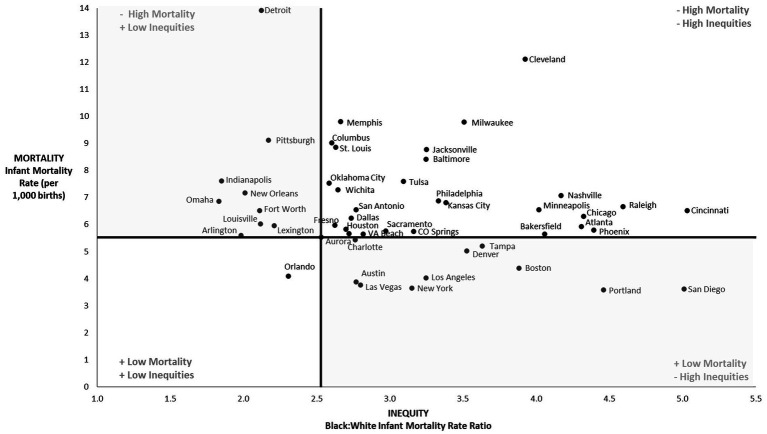
Infant mortality rates and racial equity in rates (2018–2021).

In Figure 2, we plotted the 48 cities based on both overall infant mortality rate (y-axis) and the racial inequity in infant mortality, as measured by the rate ratio (x-axis). The US infant mortality rate and Black:white inequity in infant mortality were used to separate outcomes into quadrants. The lower left quadrant represents the “best-performing cities” that had infant mortality rates below the US rate and Black:white inequity below the national level of inequity. The only city that met these criteria was Orlando. The upper right quadrant represents the “worst-performing” cities with infant mortality rates higher than the US and levels of racial inequity greater than the US. There were 27 cities with poor outcomes for both measures which met these criteria. The remaining cities performed comparatively well for either overall infant mortality or equity. For example, San Diego had one of the lowest infant mortality rates, but the largest racial inequity. Conversely, Detroit was one of the most equitable cities for infant mortality, but it also had the highest overall infant mortality rate.

**Figure 3:**
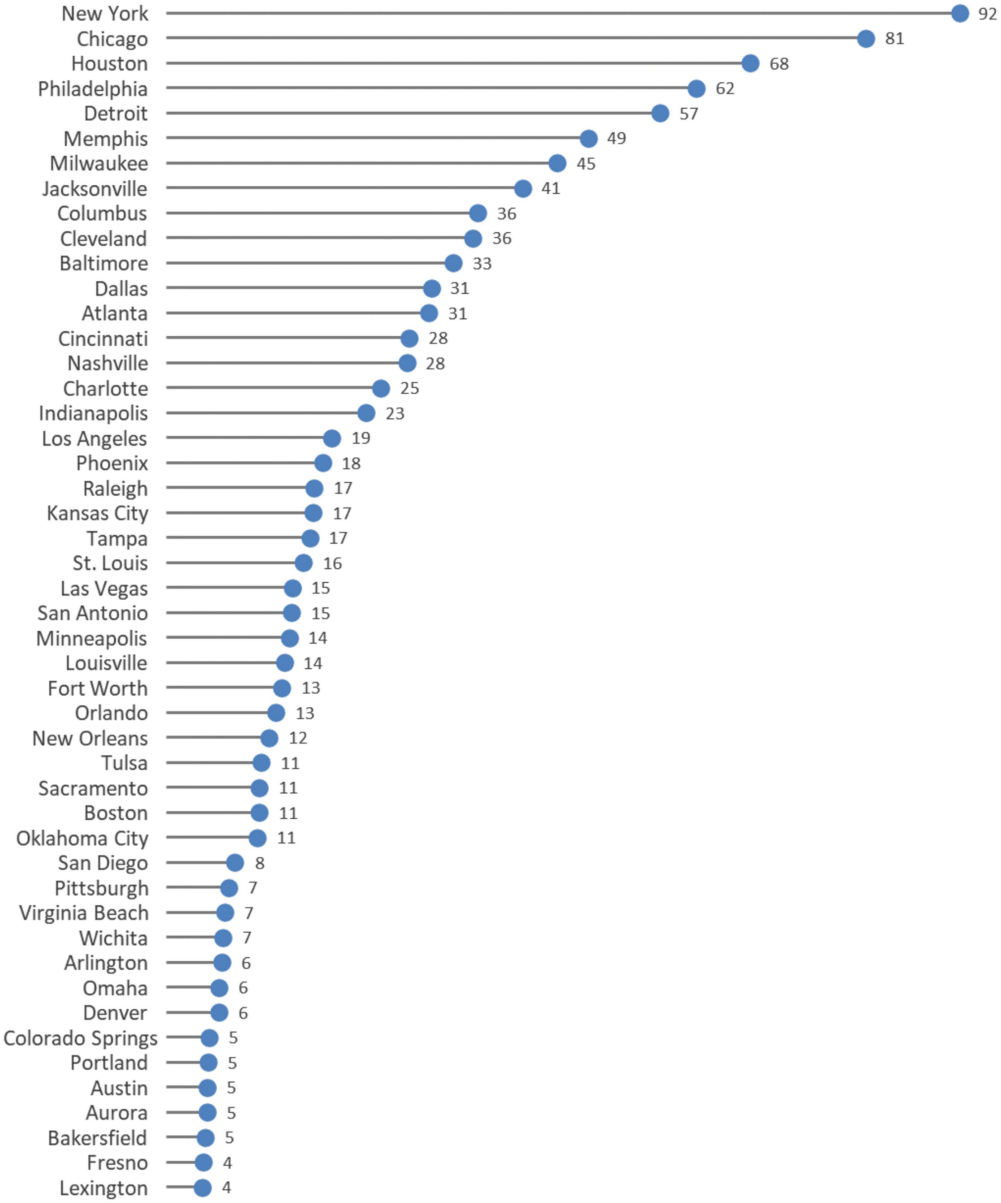
Annual excess Black infant deaths by city (2018–2021).

Figure 3 shows the annual number of excess Black infant deaths. The size of the excess Black deaths measure is a function of both the size of the Black:white inequity in a city and the size of the Black population. Excess Black infant deaths ranged from fewer than five deaths per year in seven cities to 92 deaths per year in New York. Five cities had over 50 excess Black infant deaths each year (Detroit, Philadelphia, Houston, Chicago, and New York).

### Infant mortality and measures of structural racism

Table 1 shows correlation analyses between city-level measures of sociodemographic characteristics, infant mortality rates, and Black:white rate ratios. Several city-level measures were not related to Black:white rate ratios, including education, income inequality, health insurance, rent burden, and the standardized structural racism score. None of the included measures of structural racism were significantly associated with Black:white rate ratio. In contrast, there were significant relationships between a city’s overall infant mortality rate and poverty (R^2^ = 0.55), median household income (R^2^ = −0.76), incarceration rates (R^2^ = 0.43), segregation (R^2^ = 0.37), and racial and ethnic diversity (R^2^ = −0.38).

**Table 1:** Correlations between city-level characteristics, infant mortality rates (IMR), and Black:white rate ratios (2018–2021).

			Overall IMR		Black IMR		White IMR		Black:white Rate Ratio	
	Median	Range	Coefficient^1^	*p*-value	Coefficient^1^	*p*-value	Coefficient^1^	*p*-value	Coefficient^1^	*p*-value
*Socio-demographic characteristics*										
High school graduation rate (%)^2^	88.2	78.4–94.5	−0.06	0.677	−0.03	0.851	−0.07	0.641	0.06	0.665
Black rate	88.4	74.7–93.8	**−0.35**	**0.013**	−0.03	0.824	0.01	0.943	−0.02	0.889
White rate	95.1	84.4–98.6	**−0.53**	**<0.001**	**−0.44**	**0.002**	**−0.46**	**<0.001**	0.21	0.161
Poverty rate (%)^2^	16.1	7.8–31.8	**0.55**	**<0.001**	**0.31**	**0.034**	0.22	0.130	−0.02	0.891
Black rate	24.8	14.2–37.8	**0.51**	**<0.001**	**0.38**	**0.008**	0.27	0.059	−0.02	0.891
White rate	10.2	5.7–30.6	**0.54**	**<0.001**	**0.46**	**<0.001**	**0.38**	**0.008**	−0.09	0.534
Median household income ($)^2^	61,441	33,678-89,457	**−0.76**	**<0.001**	**−0.46**	**0.001**	**−0.59**	**<0.001**	**0.33**	**0.024**
Black median	41,029	25,351-61,984	**−0.63**	**<0.001**	**−0.38**	**0.007**	**−0.39**	**0.007**	0.15	0.305
White median	77,543	44,089-116,299	**−0.73**	**<0.001**	**−0.65**	**<0.001**	**−0.73**	**<0.001**	**0.37**	**0.009**
Uninsured rate (%)^2^	10.1	3.4–23.8	0.01	0.934	−0.02	0.886	0.14	0.345	−0.20	0.176
Black rate	10.4	4.4–19.9	0.13	0.394	0.06	0.686	0.27	0.068	**−0.29**	**0.046**
White rate	6.1	2.0–11.3	0.26	0.079	0.19	0.194	**0.41**	**0.004**	**−0.34**	**0.019**
Households with high rent burden (%)^2^	40.8	34.8–52.6	−0.18	0.232	−0.21	0.145	−0.15	0.307	−0.04	0.810
Adults 18–64 years in correctional facilities (%)^3^	0.5	0–2.06	**0.43**	**0.002**	**0.35**	**0.016**	0.26	0.075	−0.01	0.921
Black rate	1.0	0–3.36	0.10	0.514	0.20	0.174	0.08	0.572	0.07	0.656
White rate	0.4	0–1.58	**0.42**	**0.003**	**0.43**	**0.002**	**0.36**	**0.013**	−0.10	0.490
Composite measures and indexes										
GINI coefficient^2^	0.5	0.42–0.57	0.05	0.725	−0.21	0.146	−0.26	0.074	0.14	0.357
Neighborhood Racial and Ethnic Segregation^4^	20.5	8.4–41.9	**0.37**	**0.010**	0.02	0.870	−0.03	0.859	0.05	0.722
Racial and Ethnic Diversity^4^	73.9	49.6–92.4	**−0.38**	**0.007**	**−0.33**	**0.023**	**−0.41**	**0.004**	0.19	0.197
Standardized Structural Racism Score^5^	0.6	−0.75-2.88	−0.04	0.818	−0.24	0.116	−0.27	0.082	0.16	0.313

1 Spearman’s rank correlation coefficient. Significant correlations (*p* < 0.05) are bolded.

2 American Community Survey 5-year estimates for 2017–2021.

3 2020 U.S. Census.

4 City Health Dashboard (15). Neighborhood Racial and Ethnic Segregation—a higher score = more segregation; Racial and Ethnic Diversity—a higher score = more diversity.

5 Siegel et al. (27). Combines 5 dimensions of structural racism (segregation, employment, economic status, education, and incarceration) into a single measure. Data not available for 5 cities: Arlington, TX; Baltimore, MD; Columbus, OH; Lexington, KY; Orlando, FL.

Similar associations were seen for the Black infant mortality rate. The white high school graduation rate (R^2^ = −0.44), the Black poverty rate (R^2^ = 0.38) and white poverty rate (R^2^ = 0.46), and median household income (overall: R^2^ = −0.46, Black: R^2^ = −0.38, white: R^2^ = −0.65) were significantly related to Black infant mortality. Additionally, the overall percentage of incarcerated adults (R^2^ = 0.35) and the percentage of white adults incarcerated (R^2^ = 0.43) were related to infant mortality. Only the Racial and Ethnic Diversity measure was significantly related to the Black infant mortality rate (R^2^ = −0.33).

The white infant mortality rate was significantly associated with the white high school graduate rate (R^2^ = −0.46), the white poverty rate (R^2^ = 0.38), and median household income (overall: R^2^ = −0.59, Black: R^2^ = −0.39, white: R^2^ = −0.73). The white uninsured rate (R^2^ = 0.41) and the White incarceration rate (R^2^ = 0.36) were each moderately associated with the white infant mortality rate. The only structural racism measure that was related to white infant mortality was the Racial and Ethnic Diversity measure (R^2^ = −0.41).

Finally, both overall median household income (R^2^ = 0.33) and white median household income (R^2^ = 0.37) were associated with the Black:white infant mortality rate ratio. Additionally, the Black uninsured rate (R^2^ = −0.29) and white uninsured rate (R^2^ = −0.34) were significantly related to the Black:white rate ratio. Interestingly, none of the included measures of structural racism were related to the Black:white infant mortality rate ratio.

## Discussion

These results show greater variation in race-specific infant mortality rates and inequities at the city level compared to analyses utilizing larger geographic levels of analysis (3). Similar to state-level analysis conducted by Matthews et al. (3), we found that cities with the largest overall infant mortality rates and race-specific rates were in the midwestern and southern parts of the US; however, inequities in infant mortality were largest in some western cities, including San Diego, Portland, Phoenix, and Bakersfield, in addition to midwestern and southern cities. The geographic variation in infant mortality rates as well as inequities in infant mortality suggests that there may be other factors associated with infant mortality and which vary by city.

We examined the correlation between city-level measures of structural racism to understand the ways in which differing levels of resources and opportunities impact rates of infant mortality across US cities. In addition to evaluating measures specific to individual domains of structural racism (high school graduation, poverty, median household income, insurance coverage, high rent burden, and incarceration), we also assessed composite scores (GINI Coefficient, Neighborhood Racial and Ethnic Segregation, Racial and Ethnic Diversity, and the Standardized Structural Racism Score) designed to examine inequality, segregation, or structural racism, which have been associated with poor health outcomes in the literature.

Similar to other studies examining the relationship between structural racism and adverse health outcomes, we found significant correlations between infant mortality rates and city-level indicators of socio-economic status (10, 11, 16). In our study, city-level poverty rate was positively associated with both overall infant mortality rates and Black infant mortality rates. This finding echoes those of Wallace et al. (11) and Bishop-Royse et al. (17), whose studies showed that increased inequity in socioeconomic measures was associated with increased rates of overall infant mortality and Black infant mortality. Our results also align with the current literature, which clearly shows a relationship between disproportionate incarceration rates of Black residents and adverse maternal and neonatal outcomes (18–20).

While others have shown associations between maternal and neonatal outcomes and educational attainment, housing, medical care, community infrastructure, and segregation (9, 21), we did not observe significant relationships between infant mortality rates and these measures in our analyses. These null findings could be attributed to the fact that our study used city-level measures rather those calculated at smaller geographic units like census tract, block group, or individual level, which allows for more specificity and greater variation.

Additionally, we detected a significant relationship between median household income, white household income and the Black:white infant mortality rate ratio. Across the other measures of structural racism, there were no statistically significant findings when using the Black:white infant mortality rate ratio as the outcome of interest. We suspect that this is a result of the associations of structural racism on Black infant mortality rates and white infant mortality rates; while increases in measures of structural racism result in negative outcomes for Black infants, in our study they were also associated with negative outcomes for white infants, resulting in null findings for the comparative measure.

Confirmation of associations between composite measures of structural racism and Black:white infant mortality rate ratios was hindered by the number of cities included in our analysis. Despite its status as a useful indicator of population health, in the United States, infant death is a rare event, such that many of our study cities could not be included because the number of infant deaths was too low for inclusion (fewer than 20 deaths over the study period).

It is also possible that the measures of structural racism deployed in this study are not adequate for city-level investigations. Much of the literature that attempts to examine the impact of structural racism on infant mortality has utilized other measures such as the Index of Concentration at the Extremes, that are more readily calculated for neighborhood or community (17, 22), county (8), and state (3) levels. It may be the case that there is too much heterogeneity in the large cities included in our sample to be adequately represented by the composite measures we utilize.

### Taking action

Many initiatives have emerged to address poor maternal and child health, as well as the persistent racial inequities in infant mortality. Initiatives range in scope, timing, and level of focus. Some seek to immediately reduce infant mortality rates, such as an initiative that provides doulas to low-income women in New York through the By My Side Support Program (13). Others seek to address inequities in maternal and child health in the medium and long term, such as initiatives that develop new clinical payment models to encourage provider action and programs seeking to increase recruitment and retention of Black maternal health clinicians (23). Other upstream interventions seek to improve maternal and infant health outcomes by addressing the root causes of inequity, through activities such as developing improvements to the built environment or enhancing educational opportunities in Black communities (12).

A key component of interventions that can successfully address inequities in birth outcomes is the involvement of local communities in understanding the history of the area, identifying problem areas, and developing interventions or programs to affect change. One program developed by CityMatCH, an organization made up of Maternal and Child Health programs in city health departments, collected oral histories of individual neighborhoods by interviewing residents. The oral history project revealed events that drastically altered life in the cities, such as new highway development or street closures that broke apart neighborhoods and caused increased isolation for residents; these types of events had not been previously recognized by health departments as a potential source of inequities that needed to be addressed. Another New Jersey initiative, Nurture NJ, focused on community building in order to transform one of the highest ranked states in maternal deaths and Black:white inequities into one of the safest places to give birth. The strategic trend in involving the people most affected by racial and ethnic inequities in birth outcomes aids in framing differences as a result of social determinants rather than merely individual risk and redirecting the focus of interventions to those that address social determinants. Other actions that have bolstered these initiatives include the recent movement of states and organizations to officially declare racism as a public health crisis and changes in National Institutes of Health funding in response to a recognition of the lack in funding for Black researchers (12).

## Limitations

This work has several limitations. The race and ethnicity data come from death certificates, which rely on the reporter’s observations or interviews with the decedent’s family. Fortunately, research suggests reporting for the racial and ethnic categories used here (white and Black races and Hispanic ethnicity) is highly accurate (24, 25). In addition, the analyses were limited to non-Hispanic Black and non-Hispanic white populations; we chose these populations because they are frequently used in the US to represent the extremes of privilege and marginalization (26) in the context of health inequities. Our study was also not able to include the Index of Concentration at the Extremes (ICE), a measure of structural racism that has been found to be associated with inequities in infant mortality in other studies (16, 17), because this measure is typically used for analyses of smaller levels of geography like neighborhoods and community areas.

### Further study

Although small numbers of deaths will make analyses challenging, additional work examining inequities affecting other racial and ethnic groups would be valuable. Further examination of racial inequity in infant mortality and its association with measures of structural racism is warranted, particularly among a greater number of geographic areas and over a longer period of time. Additionally, given the low absolute number of infant deaths in smaller geographic areas like cities, it would also be beneficial to focus research efforts on the link between measures of structural racism and outcomes that are likely to be related to infant mortality, such as preterm birth, low birthweight, and maternal access to prenatal care.

## Conclusion

Mortality in the U.S. continues to depend on both race and geography, reflecting the long-term effects of racism on health, as well as the importance of looking at population-level factors. Here we highlight racial inequities in infant mortality at the city level and explore the social and structural characteristics of cities that may underlie those disparities. Addressing the appalling levels of inequity within this critical health outcome will require both individual- and population-level interventions, at the local and national levels. This study provides valuable data to help understand current city-level inequities to guide urban policies and programs aimed at improving birth outcomes.

## Data Availability

The data analyzed in this study is subject to the following licenses/restrictions: the analysis used restricted use mortality and natality datasets from the National Center for Health Statistics. Researchers may submit a data request for the datasets, which includes analytical plans and is subject to approval by the National Center for Health Statistics. Requests to access these datasets should be directed to nvssrestricteddata@cdc.gov.
