# Supplementary material for: Black:white inequities in infant mortality across the 69 most populous US cities, 2018–2021
Source: Front Public Health. 2025 Feb 26;13:1484433. doi: 10.3389/fpubh.2025.1484433 (PMC11897040; doi:10.3389/fpubh.2025.1484433)
Supplement: Supplementary file 1 [file Supplementary_file_1.pdf]

Supplemental Table 1. City-Level Measures of Socio-Demographic Characteristics and Structural Racism

	High School Degree (%)[1]			Below Poverty Line (%)[1]			Median Household Income ($)[1]			Uninsured (%)[1]			Adults in Jail (%)[2]			High Rent Burden (%)[1]	GINI Coeffic-ient[1]	Racial and Ethnic Segregation[3]	Racial and Ethnic Diversity[3]	Structural Racism Score[4]
City	All	Black	White	All	Black	White	All	Black	White	All	Black	White	All	Black	White					
United States	89	87	94	13	22	9	69,021	46,401	75,208	9	10	6	0.94	2.68	0.67	40	0.48	11.1	62.0	
Albuquerque, NM	91	94	97	16	23	11	56,366	43,192	64,408	8	8	4	0.03	0.08	0.02	43	0.47	13.2	67.3	
Anaheim, CA	78	93	94	13	16	11	81,806	72,156	90,995	11	8	5	0.03	0.09	0.02	50	0.43	10.6	74.2	
Arlington, TX	85	94	95	14	16	10	65,481	54,034	80,449	20	18	11	0.00	0.00	0.00	44	0.43	13.4	85.0	
Atlanta, GA	92	86	99	19	28	7	69,164	38,854	114,195	11	15	5	2.06	3.37	0.81	41	0.57	33.1	71.0	2.88
Aurora, CO	87	90	96	10	15	6	72,052	60,133	81,002	12	11	6	0.24	0.18	0.07	49	0.42	10.8	82.2	-0.75
Austin, TX	91	90	98	13	22	8	78,965	50,114	94,027	13	14	7	0.17	0.71	0.13	37	0.48	15.9	76.0	0.66
Bakersfield, CA	82	87	93	16	29	11	69,014	42,015	84,107	8	5	4	0.16	0.12	0.06	44	0.44	17.4	70.6	0.29
Baltimore, MD	86	84	92	20	25	12	54,124	42,493	84,043	6	6	4	0.63	0.97	0.26	43	0.51	30.8	64.0	
Boston, MA	88	85	97	18	19	10	81,744	52,898	116,299	3	4	2	0.18	0.36	0.13	40	0.54	24.6	86.1	1.49
Charlotte, NC	90	91	97	12	15	6	68,367	49,566	94,578	13	12	6	0.27	0.56	0.13	37	0.50	20.0	66.1	0.52
Chicago, IL	86	86	96	17	28	9	65,781	37,816	96,025	10	9	5	0.32	0.84	0.12	39	0.53	41.9	84.5	2.04
Cincinnati, OH	89	83	93	25	34	15	45,235	29,107	65,885	7	8	5	0.60	0.94	0.48	38	0.54	23.2	66.3	0.82
Cleveland, OH	83	81	87	31	38	20	33,678	25,351	47,138	8	7	7	0.70	0.87	0.81	43	0.51	30.6	75.5	0.59
Colorado Springs, CO	94	94	97	11	15	9	71,957	54,493	76,592	8	11	6	0.50	1.56	0.43	43	0.44	8.8	62.6	-0.55
Columbus, OH	90	87	94	18	27	13	58,575	41,575	68,148	9	11	7	0.33	0.60	0.28	35	0.44	19.7	70.9	
Corpus Christi, TX	85	90	95	17	24	12	59,993	42,991	71,677	19	17	12	0.47	0.08	0.88	41	0.46	15.9	56.7	-0.12
Dallas, TX	80	88	97	18	25	8	58,231	39,396	88,607	24	20	9	0.48	1.00	0.51	39	0.53	26.9	80.4	1.36
Denver, CO	90	90	98	12	23	7	78,177	50,032	92,491	10	7	5	0.64	1.72	0.49	37	0.49	19.3	72.1	0.82
Detroit, MI	83	85	84	32	32	31	34,762	33,274	44,089	8	7	7	0.27	0.10	1.42	49	0.51	38.6	49.6	-0.25
El Paso, TX	81	96	96	18	11	11	51,325	58,505	69,666	20	12	11	0.69	1.28	1.82	41	0.47	14.9	41.9	-0.98
Fort Worth, TX	84	89	95	13	20	8	67,927	46,474	84,880	19	16	10	1.25	2.01	1.41	42	0.45	20.6	79.6	0.26
Fresno, CA	79	88	93	23	32	13	57,211	38,095	68,071	7	5	5	0.87	1.89	0.80	48	0.48	12.1	78.0	0.19
Henderson, NV	94	96	96	8	15	7	79,611	56,730	83,042	7	6	6	0.14	0.30	0.06	40	0.45	7.2	75.5	-0.65
Honolulu, HI*	93	97	98	9	7	8	92,600	74,949	96,826	4	4	3	0.43	1.02	0.49	47	0.43	12.4	79.8	
Houston, TX	80	89	97	20	25	9	56,019	40,794	92,247	24	18	8	0.66	1.24	0.67	43	0.53	23.2	83.6	1.23
Indianapolis, IN*	87	86	92	16	24	11	54,601	38,242	65,451	10	10	7	0.26	0.39	0.28	41	0.47	21.6	73.9	0.23

Irvine, CA	96	87	98	12	16	10	114,027	101,624	113,844	5	3	3	0.00	0.00	0.00	43	0.47	6.2	76.5	-0.86
Jacksonville, FL	90	88	93	15	22	11	58,263	42,972	68,372	12	12	10	0.57	1.02	0.48	41	0.47	17.1	77.2	-0.16
Kansas City, MO	91	89	95	15	25	9	60,042	37,182	73,690	12	14	8	0.44	0.48	0.40	38	0.47	23.1	69.2	0.69
Las Vegas, NV	85	88	94	15	27	10	61,356	35,933	73,301	13	10	7	0.36	1.34	0.29	46	0.49	12.5	82.1	0.12
Lexington, KY*	92	89	95	16	29	12	61,526	39,438	68,447	7	9	4	1.10	2.91	0.88	39	0.49	13.0	61.0	
Long Beach, CA	81	91	96	15	21	10	71,150	49,495	91,095	9	8	4	0.07	0.23	0.06	45	0.46	14.8	84.1	0.35
Los Angeles, CA	78	89	96	17	24	10	69,778	44,614	95,368	11	7	5	0.30	1.03	0.20	49	0.52	26.0	79.7	0.85
Louisville, KY*	91	89	93	14	25	10	61,633	41,512	69,284	5	6	4	0.44	0.79	0.41	35	0.47	21.0	61.9	-0.09
Memphis, TN	87	86	95	24	29	11	43,981	36,046	66,892	14	14	9	1.35	1.28	1.58	47	0.53	31.0	62.3	0.84
Mesa, AZ	90	95	95	12	15	8	65,725	52,898	69,933	12	10	7	0.01	0.03	0.00	42	0.43	12.3	64.6	
Miami, FL	79	76	96	21	34	10	47,860	29,403	99,911	19	21	9	0.81	3.36	0.84	53	0.56	34.2	56.0	3.49
Milwaukee, WI	85	85	94	24	32	13	45,318	31,959	61,182	9	7	5	0.32	0.55	0.25	43	0.47	35.0	81.9	0.88
Minneapolis, MN	91	75	98	17	35	10	70,099	30,617	83,900	6	7	4	0.28	1.09	0.11	36	0.49	17.3	73.3	2.67
Nashville, TN*	90	89	95	14	22	9	66,047	49,137	77,293	12	12	8	0.88	2.01	0.70	40	0.49	19.0	71.9	-0.09
New Orleans, LA	88	84	97	24	32	11	45,594	29,502	79,205	9	9	7	0.67	0.92	0.43	53	0.56	31.7	65.6	2.27
New York, NY	83	85	94	17	20	11	70,663	53,075	102,960	7	7	4	0.15	0.35	0.11	43	0.55	32.9	90.4	0.92
Newark, NJ	77	84	71	26	28	19	41,335	36,982	51,651	18	11	19	4.33	5.09	9.19	49	0.50	30.9	73.1	-0.15
Oakland, CA	85	90	97	14	19	7	85,628	53,363	132,296	7	6	3	0.06	0.20	0.03	41	0.50	16.8	94.1	1.17
Oklahoma City, OK	88	91	94	15	28	9	59,679	37,637	70,599	14	14	9	0.85	2.12	0.70	37	0.47	18.1	77.8	0.26
Omaha, NE	90	88	96	12	25	8	65,359	36,430	72,792	10	14	6	0.70	2.08	0.49	37	0.47	21.6	64.5	0.99
Orlando, FL	92	85	96	16	22	10	58,968	41,263	77,792	15	17	11	0.00	0.00	0.00	45	0.50	20.4	82.1	
Philadelphia, PA	87	87	93	23	27	12	52,649	39,352	74,279	7	7	5	0.53	0.93	0.25	43	0.52	33.3	83.1	0.70
Phoenix, AZ	84	90	95	15	22	9	64,927	48,916	76,716	15	11	7	0.86	2.14	0.99	39	0.47	21.5	73.9	0.10
Pittsburgh, PA	94	89	95	20	33	14	54,306	28,016	64,982	6	7	4	0.98	3.21	0.50	39	0.52	20.8	64.6	0.89
Portland, OR	93	89	97	13	28	10	78,476	44,172	84,513	6	7	5	0.32	1.33	0.29	41	0.47	8.4	64.4	0.95
Raleigh, NC	92	92	98	12	16	8	72,996	48,793	90,812	10	12	5	1.23	2.29	1.07	39	0.47	16.9	74.6	0.31
Riverside, CA	83	92	94	13	15	10	76,755	66,237	87,422	9	9	5	0.32	1.12	0.33	48	0.43	10.1	73.0	-0.84
Sacramento, CA	86	91	94	15	20	11	71,074	53,562	80,661	6	5	4	0.86	2.16	0.84	43	0.45	10.7	92.4	-0.41
San Antonio, TX	83	92	96	18	23	10	55,084	42,118	71,063	17	14	10	0.42	0.96	0.36	43	0.46	15.3	63.9	-0.12
San Diego, CA	89	91	98	12	20	8	89,457	56,875	101,595	7	8	4	0.27	0.54	0.26	44	0.46	19.9	83.0	0.35
San Francisco, CA	89	87	98	10	26	7	126,187	44,142	160,007	4	6	2	0.17	1.40	0.09	29	0.51	14.2	83.3	3.45
San Jose, CA	86	91	96	8	12	6	125,075	85,395	138,007	5	5	3	0.09	0.51	0.07	40	0.45	15.8	81.3	-0.04
Santa Ana, CA	63	88	95	12	10	10	77,283	75,438	94,570	15	8	6	1.56	11.06	5.94	47	0.40	22.2	55.6	-0.72

Seattle, WA	96	90	98	10	24	7	105,391	48,093	114,053	4	8	3	0.18	1.09	0.14	35	0.48	10.7	72.5	1.50
St. Louis, MO	89	83	95	20	28	11	48,751	30,947	65,322	11	13	7	0.57	0.98	0.36	40	0.49	28.7	67.4	0.99
St. Paul, MN	88	80	97	18	31	9	63,483	36,910	75,320	6	9	4	0.14	0.43	0.07	40	0.48	17.6	82.3	1.43
Stockton, CA	78	90	91	16	23	12	63,916	46,793	74,951	7	6	4	0.02	0.00	0.01	47	0.46	11.9	85.0	
Tampa, FL	88	84	95	17	28	9	59,893	36,634	82,476	11	11	8	0.48	1.10	0.40	44	0.54	20.8	81.1	1.15
Tucson, AZ	86	84	95	20	26	16	48,058	41,609	51,915	11	10	7	2.36	7.56	2.07	43	0.45	15.8	68.9	-0.69
Tulsa, OK	88	90	94	18	29	12	52,438	33,261	60,769	17	17	11	0.46	0.96	0.47	37	0.51	16.0	78.4	0.24
Virginia Beach, VA	95	93	96	8	14	6	81,810	61,984	88,105	7	8	6	0.41	1.07	0.33	38	0.43	10.7	72.9	-0.54
Washington, DC	92	88	100	15	25	5	93,547	51,562	153,969	3	4	1	0.46	1.00	0.13	36	0.52	26.9	76.9	4.28
Wichita, KS	88	88	94	15	29	10	56,374	34,744	61,987	12	15	8	0.67	2.07	0.60	36	0.47	15.3	68.9	0.42

1. Source: American Community Survey 5-year estimates for 2017-2021

2. Source: 2020 U.S. Census

3. Source: City Health Dashboard. Racial and Ethnic Segregation - a higher score indicates more segregation at the neighborhood level; Racial and Ethnic Diversity - a higher score indicates more diversity.

4. Source: Siegel et al. 2023. Combines variables encompassing 5 dimensions of structural racism (segregation, employment, economic status, education, and incarceration) into a single measure. Data not available for 5 cities: Arlington, TX; Baltimore, MD; Columbus, OH; Lexington, KY; Orlando, FL.
